# Notes on Preparations for the Sick

**Published:** 1889-12

**Authors:** 


### Notes on Preparations for the Sick.

---

**Syrup Trifolium Compound.** Parke, Davis, & Co., Detroit.

This preparation was first introduced in 1885, as being superior in efficacy to the combination of alteratives known as bamboo-brier root compound, alterative compound, or by other and copyrighted names. Experience with the latter combination led to the belief that the formula could be greatly improved, and, after much clinical investigation, this preparation was adopted as the best for general use. Prescribed in all forms of secondary syphilis, its restorative action was found prompt and unequivocal.

Each fluid ounce contains the active constituents of thirty-two grains of red clover, sixteen grains each of stillingia, burdock root, poke root, berberis aquifolium, and cascara amarga, four grains of prickly-ash bark, with eight grains of potassium iodide.

The British Pharmacopœia is singularly poor in vegetable alteratives such as are contained in this elaborate compound. English physicians have, to some extent, relied on sarsaparilla, but have for the most part prescribed it in combination with more active drugs, which have undoubted efficacy.

If there be any value in vegetable alteratives, such a combination as the above ought to have a very wide range of utility in proportion to the number of drugs which it contains; and, if the vegetable ingredients fail, there is still one potent factor, in which all medical men have faith, and to which the efficacy of the compound is probably mainly due.

It may be said that the **Syrup Trifolium Co.** is an unscientific compound: it certainly is in opposition to the usual modern type of simplicity in prescribing; but, on the other hand, we must accept the evidence of much experience that combinations like this are more efficacious than a simple solution of the iodide of potassium in the same quantity.

Again, iodide of potassium is a drug which requires some vehicle to disguise its taste; and further, it is often advantageous to prescribe it in such a form that the patient may not identify its name.

The combination will serve various purposes of this kind; and, if not strong enough, can easily be fortified by the addition of more of the iodide. We are informed that there is scarcely a condition requiring alterative treatment in which syrup trifolium compound, alone or combined with other agents, may not be used to advantage.

---

**The Universal Digestive Tea.** The Universal Digestive Tea Company Limited, Manchester.

This is prepared upon a new principle, by which the tannin is entirely neutralised and the injurious oils to a great extent extracted, while the fine delicate flavour of the blend of India, China, and Ceylon teas is retained.

Those who wish to have tea without tannin will doubtless prefer this preparation to any other: it is commonly believed that tannin is a very injurious substance, and that tea-drinkers' dyspepsia is due to this ingredient. It is found that persons who suffer considerably from unprepared tea can take this "**Digestive Tea**" without any ill effects; and no matter how long it stands, it does not become bitter or injurious. We have found its flavour to be agreeable, and its effects to be in all respects satisfactory. The following table shows the effect on the digestion of farinaceous food of two lots of tea, both taken from the same tea estate, and at the same time of the year—one lot dried upon the old system, and the other on the system of the Universal Digestive Tea Company (Limited). An equal strength of infusion was made from each of these teas. The first column shows the varying percentage of the tea infusion used. The second and third columns show the time occupied in digesting the food. One column ordinary tea, and the other "Universal Digestive Tea":

| Percentage of Infusion. | Time occupied in digesting with Digestive Tea. | Time occupied in digesting with Ordinary Tea. |
|---|---|---|
| 2 per cent. | 5 minutes. | 28 minutes. |
| 3 " " | 5 " | 50 " |
| 5 " " | 5 " | 190 " |

The starch was converted into grape sugar as quickly when digestive tea was added as if pure water only had been used; whereas, when five per cent. of ordinary tea was used, digestion was delayed for 190 minutes, or three hours and ten minutes.

This speaks for itself.

---

**"Tasteless" Castor Oil.**—Allen & Hanburys, London.

Castor oil is a drug which has not yet been, and is not likely to be, altogether supplanted by its more modern rivals; nevertheless, it has been found that patients often decline to take it, and choose some more palatable but less efficient substitute. Modern improvements have accordingly been introduced, by which the only drawback has been removed, and this, "the safest, surest, and most generally valuable aperient known," may be given absolutely free from odour and unpleasant taste. The activity of the oil is in no degree reduced by the new method of preparation.

The best way of taking Allen and Hanburys' **Castor Oil** is thoroughly to mix the dose with about four times as much hot milk,—this is most effectually accomplished by shaking the two together in a bottle which they do not more than half fill. The mixture should then be taken immediately. The oil may also be taken floating on coffee, brandy, lemon-juice, or peppermint water, or in any of the ordinary ways; but, when taken as above directed, its activity appears to be increased, and, being rendered very limpid by the hot milk, its oily nature is not perceived. Children take it very readily in this form, in which, indeed, it is scarcely distinguishable from rich milk.

---

**Liq. Papain et Iridin Co.**—Seymour, Hamilton, & Co., London.

This combination of two useful drugs is well spoken of by those who have tried it. Papain, as a digestive, appears to be worthy of ranking with pepsine, and iridin, as a cholagogue, ranks near to euonymin. The mixture is therefore an energetic peptonising agent, and a powerful hepatic stimulant.

Papain should be preferred to pepsine, as being entirely vegetable in its origin, and not prepared, like the other ferments, from the stomachs of pigs and such animals, with always a possible doubt as to the freshness or healthy condition of the material, which has been proved to contain bacteria, bacilli, &c., which will inevitably be also present in the pepsine manufactured from it. (The existence of what are known as ptomaines, or cadaveric alkaloids, has now been established beyond dispute.)

"Iridin is justly," says Dr. Glover Coe, "esteemed as one of our most valuable remedies. It is eminently resolvent, and exercises a marked influence over the whole glandular system, quickening the activity of the secreting apparatus, and promoting depuration."

One drachm diluted, half an hour after each meal, gives relief in cases where there is either deficient secretion of the peptic ferments, abnormal fermentation, or a combination of both conditions.

